# Expanding the Genomic Landscape of HBOC and Cancer Risk Among Mutation Carriers

**DOI:** 10.3390/ijms26135928

**Published:** 2025-06-20

**Authors:** Maria Teresa Vietri, Chiara Della Pepa, Gemma Caliendo, Alessia Mignano, Luisa Albanese, Marialaura Zitiello, Marianna Stilo, Anna Maria Molinari

**Affiliations:** 1 Department of Precision Medicine, University of Campania “Luigi Vanvitelli”, S. Andrea delle Dame, Via L. De Crecchio, 7, 80138 Napoli, Italy; 2 Unit of Clinical and Molecular Pathology, AOU University of Campania “Luigi Vanvitelli”, S. Andrea delle Dame, Via L. De Crecchio, 7, 80138 Napoli, Italy; 3 Oncology Unit, Vallo Della Lucania-Agropoli Hospital, ASL Salerno, Via F Cammarota, 84078 Vallo Della Lucania, Italy

**Keywords:** hereditary breast and ovarian cancer (HBOC), multigene panel testing, bioinformatic analysis, homologous recombination (HR) pathway, cancer susceptibility genes

## Abstract

Hereditary breast and ovarian cancer (HBOC) syndrome is primarily associated with mutations in BRCA1 and BRCA2, but increasing evidence links it to other malignancies, including male breast, prostate, and pancreatic cancers. Advances in genetic testing have led to the use of multigene panels, revealing that additional genes contribute to HBOC risk. We tested 280 patients with suspected HBOC using a multigene panel including BRCA1, BRCA2, and other genes involved in homologous recombination (HR) and additional DNA repair mechanisms. Variants were classified as pathogenic variants (PVs), variants of uncertain significance (VUS), or novel. In silico tools were used to predict the clinical relevance of VUS and novel variants. The clinical phenotype of families carrying a PV was evaluated. PVs were identified in 19.3% of patients: 8.9% in BRCA1/2 and 10.4% in other genes, mainly CHEK2, ATM, PALB2, and BRIP1. An additional 1.8% of cases harbored likely pathogenic VUS or novel variants according to bioinformatic prediction. Breast and ovarian cancer were the most frequent malignancies in our population, both in the BRCA group and in those with PVs in other susceptibility genes. Broad genetic testing beyond BRCA improves HBOC diagnostics, supports identification of at-risk families, and enables more personalized surveillance and treatment.

## 1. Background

Pathogenic variants (PVs) in BRCA1 and BRCA2 are causative of hereditary breast and ovarian cancer (HBOC), a hereditary syndrome associated with increased lifetime risk of breast cancer (BC) and ovarian cancer (OC) [1]. Individuals suffering from HBOC are also at risk of developing male BC [2], prostate cancer [3], pancreatic cancer [4,5], colorectal cancer CRC [6,7], and melanoma [8]. The precise mechanism through which mutations in BRCA genes lead to HBOC has been partially explained thanks to PARP inhibitors (PARPis), a group of anticancer molecules able to induce synthetic lethality in BRCA-mutated cells and hence in HBOC patients with BC and/or OC. PARP inhibition works well in hereditary BC/OC due to the deficit of the homologous recombination (HR) pathway observed in these patients as a result of their BRCA mutation [9]. Variants in genes other than BRCA1 and BRCA2, though still integral to the homologous recombination (HR) repair pathway, have also been found in the HBOC syndrome. However, the clinical features of the condition, such as the type of cancer, age of onset, and lifetime risk, vary significantly depending on the specific gene affected.

For instance, PVs in PALB2 are linked to an increased risk of BC, though the magnitude of risk is slightly lower than that conferred by BRCA1/2 (lifetime risk approximately 35–53% versus 45–72% for BRCA). In addition to BC, PALB2 mutations may also confer a modestly increased risk of ovarian, pancreatic, and possibly male BC [10]. Conversely, mutations in ATM and CHEK2 are generally associated with moderate penetrance, with a lifetime BC risk of about 25–33% for ATM and 28–37% for CHEK2. CHEK2 has also been linked to an increased risk of contralateral BC and, in some studies, CRC, and prostate cancer, while ATM mutations may confer a slightly increased risk of pancreatic and possibly OC [11,12,13].

This gene-specific variability influences not only risk assessment but also clinical decision-making, including screening intervals, risk-reducing strategies, and eligibility for targeted therapies. The advent of Next Generation Sequencing (NGS) and the widespread use of multigene panels are rapidly expanding the spectrum of genes implicated in HBOC. This technological progress is reshaping our understanding of the syndrome and raising important questions about the most appropriate surveillance and management protocols. As the clinical significance of each gene becomes better defined, there is a growing movement toward personalized risk stratification, where surveillance and prevention strategies are increasingly tailored to the individual’s genetic profile.

Furthermore, in patients with advanced-stage cancer, the identification of a germline variant in an HR-related gene may indicate sensitivity to PARP inhibition, and promising data have already been published [14]. This not only broadens the spectrum of available treatment options but also highlights the clinical relevance of comprehensive genetic testing in both preventive and therapeutic decision-making.

However, while panel testing enables access to a broader range of genomic data, it has also led to a substantial increase in the number of variants of uncertain significance (VUS) identified in routine diagnostics. Interpreting the clinical significance of these VUS remains one of the most critical challenges for oncologists and genetics professionals.

This study focuses on HBOC syndrome caused by variants in genes other than BRCA1 and BRCA2, describing the most frequently affected genes as well as the clinical features observed in patients and their families.

We also identified several VUS and novel variants in cancer susceptibility genes. These variants were analyzed using bioinformatic prediction tools to perform in silico assessments aimed at evaluating their potential clinical significance.

## 2. Results

### 2.1. Mutation Rate and Affected Genes

**Figure 1:**
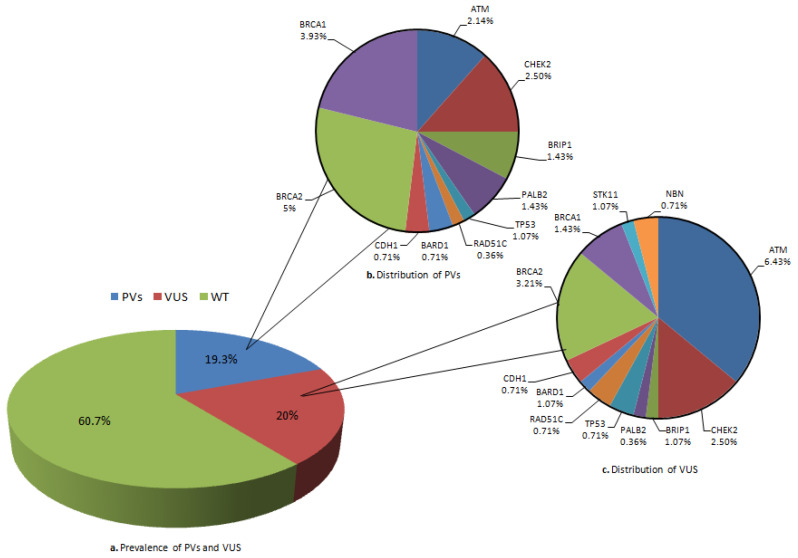
(**a**) Prevalence of PVs and VUS among cancer susceptibility genes; (**b**) distribution of PVs; (**c**) distribution of VUS. Legend: PVs, pathogenic variants; VUS, variants of uncertain significance; WT, wild type.

We evaluated 280 individuals with suspected HBOC syndrome; the median patient age at the time of the test was 57 years old (range 21–89). We found that 19.3% of them (54/280) had a PV in cancer susceptibility genes and 20% (56/280) carried a VUS (Figure 1a).

Among the patients with a PV, 8.9% (25/280) had a BRCA mutation and 10.4% (29/280) had a PV in other genes included in the panel.

Specifically, 3.93% (11/280) harbored a PV in BRCA1, 5% (14/280) in BRCA2, 2.50% (7/280) harbored a PV in CHEK2, 2.14% (6/280) in ATM, 1.43% (4/280) in BRIP1, 1.43% (4/280) in PALB2, 1.07% (3/280) in TP53, 0.36% (1/280) in RAD51C, 0.71% (2/280) in BARD1, and 0.7% (2/280) in CDH1. We did not find any PV in STK11, PTEN, or in RAD51D (Figure 1b).

Among the 56 probands carrying a VUS, 6.43% (18/280) involved ATM, 3.21% (9/280) BRCA2, 2.50% (7/280) CHEK2, 1.43% (4/280) BRCA1, 1.07% (3/280) BRIP1, 1.07% (3/280) STK11, 1.07% (3/280) BARD 1, 1.07% (3/280) CDH1, 0.71% (2/280) TP53, 0.71% (2/280) NBN, 0.71% (2/280) RAD51C, 0.36% (1/280) PALB2; we did not find any VUS in PTEN and in RAD51D (Figure 1c).

Notably, we identified for the first time the CDH1 novel mutation c.1678_1688del (p.Thr560Profs*24). This variant involves the deletion of 10 nucleotides at positions 1678 to 1688, leading to a frameshift starting at amino acid threonine 560. The frameshift alters the amino acid sequence, resulting in the insertion of a proline and the creation of a premature stop codon (Ter) 24 amino acids downstream.

Besides, we found 28 novel variants, not reported in any database, in 32 patients out of 280 (11.4%). 1.8% (5/280) carried a novel variant in ATM, 1.8% (5/280) in CHEK2, 1.8% (5/280) in BARD1, 1.4% (4/280) in PTEN, 0.7% (2/280) in TP53, 0.7% (2/280) in PALB2, 0.7% (2/280) in BRCA1, 0.3% (1/280) in BRCA2, 0.3% (1/280) in RAD51C, and 0.3% (1/280) in BRIP1.

### 2.2. In Silico Analysis

The results of the in silico analysis of the VUS are described in Supplementary Table S1 (available in the Supplementary Material). Out of 46 VUS found in 56 patients, 6 VUS were classified as likely pathogenic (LP) class II; 11 remained of uncertain significance, class III; 7 were classified as likely benign, class IV; and 22 benign, class V.

The findings of the in silico analysis of the novel variants are detailed in Supplementary Table S2 (available in the Supplementary Material). At the bioinformatic analysis, 27 novel variants out of 28 resulted in benign, class V; 1 novel variant in BARD1, c.21889A>G (p.Glu730Pro), resulted of uncertain significance.

Therefore, 1.8% (5/280—one patient harbored 2 LP variants) of patients carried an LP variant, based on the results of the prediction tools.

### 2.3. Clinical Features of Patients and Families

The clinical features observed in patients with a BRCA mutation and in their families are summarized in Table 1: 17 patients had BC, 2 had OC, 2 has prostate cancer, 2 had gastric cancer, 1 had lung cancer, 1 had laryngeal cancer, and 1 had CRC.

The clinical phenotype noted in patients harboring a PV in the other genes and in their families is shown in Table 2: 16 patients had BC, 4 OC, 1 bilateral BC, 2 BC and OC, 1 prostate cancer, 1 pancreatic cancer, and 1 melanoma. Moreover, 1 patient was diagnosed with CRC, 1 with lung cancer, and 1 with gastric cancer.

Genetic testing of patients’ family members using sanger sequencing was performed in 39 out of 54 cases with PVs, resulting in the analysis of 71 additional individuals, 22 of whom were found to carry the proband’s variant.

**Figure 2:**
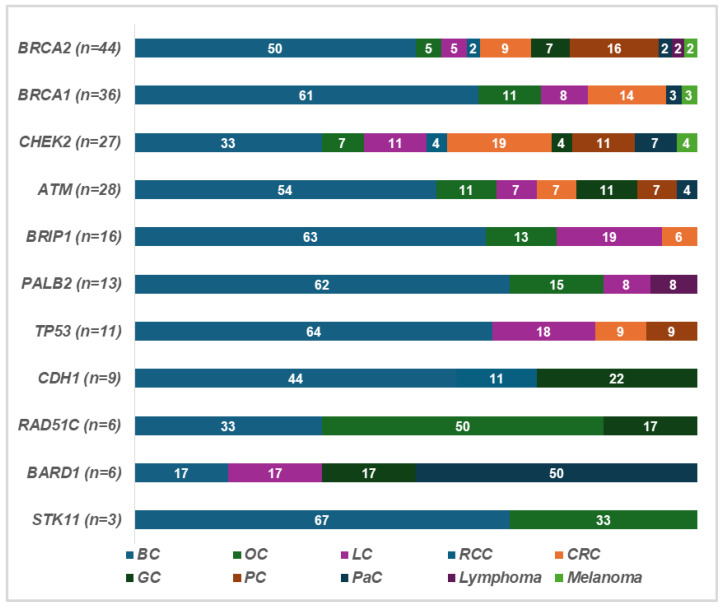
Cancer types among patients with PVs and their families. The numbers indicate the percentage of patients diagnosed with the specific tumor type among the patients harboring a pathogenic variant in BRCA1/2 or in other cancer susceptibility genes and their families. Abbreviations: BC, breast cancer; OC, ovarian cancer; LC, lung cancer; RCC, renal cell cancer; GC, gastric cancer; PC, prostate cancer; PaC, pancreatic cancer.

The different types of cancer observed in patients carrying a PV in cancer susceptibility genes and in their families are shown in Figure 2: we observed a high prevalence of BC and OC. CRC often recurs, particularly in patients with CHEK2 mutations, while gastric cancer was found to be frequent in individuals with CDH1 mutations.

## 3. Discussion

Our data indicate that multigene panel testing identifies a PV in a cancer susceptibility gene in 19.3% of cases. Including variants classified as ‘likely pathogenic’ through in silico analysis increases this proportion by an additional 1.8%, resulting in a total of 21.1% of patients with a detectable deleterious variant associated with the disease. The percentage of patients in whom a causative mutation was identified is consistent with, and even higher than, that reported in previous studies, which documented mutation rates in similar populations ranging from 16.1% to 20.7% [16,17].

The multigene panel we used includes genes directly involved in the homologous recombination (HR) pathway, such as BRCA1, BRCA2, PALB2, RAD51C, RAD51D, ATM, BRIP1, and BARD1; others, such as CHEK2 and NBN, part of broader DNA damage response pathways (e.g., checkpoint signaling), which support genomic stability but are not strictly HR genes; and finally, genes such as TP53 (Li-Fraumeni syndrome), PTEN (Cowden syndrome), STK11 (Peutz-Jeghers syndrome), and CDH1 (hereditary diffuse gastric cancer syndrome) that are associated with hereditary cancer syndromes that confer an elevated risk of BC, along with predisposition to other tumor types.

In recent years, the use of multigene panels for HBOC diagnosis has revealed that CHEK2 and ATM are among the most frequently mutated genes after BRCA1 and BRCA2 [17,18,19,20]. In our cohort, we also observed an equal number of PVs in PALB2 (which emerges as one of the most commonly altered genes) and in BRIP1.

The clinical features seen in our patients confirm a high prevalence of BC and OC in individuals with PVs in BRCA1/2, ATM, BRIP1, PALB2, and RAD51C, which are genes closely involved in the HR pathway and already largely associated with HBOC [21,22,23].

Besides, in our cohort, we observed a relatively high number of lung and gastric cancer cases.

Lung cancer is not typically associated with the HBOC syndrome, as highlighted in a recent meta-analysis [1]. Environmental factors such as smoking or other genetic predispositions may account for this finding, although a potential association with specific BRCA2 variants has been reported [24,25].

With regard to gastric cancer, most cases in our cohort were observed in families carrying PVs in BRCA2 and CDH1. Over the years, growing evidence has supported an increased gastric cancer risk associated with BRCA2 PVs [26]. The predisposition is even stronger in carriers of CDH1 PVs, as this gene is linked to the Hereditary Diffuse Gastric Cancer (HDGC) syndrome, an autosomal dominant disorder characterized by a lifetime risk of advanced gastric cancer of approximately 10.3% in males and 6.5% in females. Notably, germline CDH1 PVs also cause increased risk of BC, estimated as a lifetime risk of 36.8% in the recent study from Ryan et al. [27].

With reference to CHEK2, following the large study by Mundt et al. [13], which reported no increased risk of CRC in individuals with germline CHEK2 pathogenic variants, the NCCN guidelines [28] have withdrawn the recommendation for colonoscopy screening in these patients, unless there is a significant family history of CRC. However, in our series, including 7 patients with CHEK2 PVs and 1 patient with an LP variant in CHEK2, we found 3 cases of CRC. It is noteworthy that one of these patients carried the CHEK2 variant I157T (patient 13 in Table 2, variant indicated as “c.470T>C (p.lle157Thr)”), which has been traditionally associated with CRC risk [29].

Interestingly, we also identified pancreatic and lung cancer in two patients carrying PVs in BARD1. Germline PVs in BARD1 have been implicated in increased risk for early-onset BC, especially triple negative, neuroblastoma, and mesothelioma [30,31,32]. There appears to be no established association between BARD1 and ovarian, pancreatic, or lung cancer; however, larger studies are needed to confirm these findings.

According to current evidence, the NCCN guidelines recommend considering risk-reducing mastectomy (RRM) not only for BRCA1/2 mutation carriers but also for individuals with germline mutations in TP53, PTEN, CDH1, STK11, and selectively for PALB2, CHEK2, and BARD1. For TP53, PTEN, CDH1, and STK11 mutation carriers, the markedly increased lifetime risk of BC supports the discussion of preventive mastectomy. In carriers of PALB2, CHEK2, and BARD1, RRM is not routinely recommended but may be considered on an individualized basis, particularly in the presence of a personal or family history of BC.

Risk-reducing salpingo-oophorectomy (RRSO) is recommended for carriers of RAD51C, RAD51D, BRIP1, and STK11, in addition to BRCA1/2 mutation carriers, generally between the ages of 45 and 50 years, depending on the specific gene and individual risk assessment. For carriers of ATM, CHEK2, and BARD1 mutations, enhanced breast surveillance (including annual breast MRI and mammography) is preferred. Preventive surgery is not routinely indicated but may be discussed in the context of significant family history or patient preference. No RRSO is currently recommended for ATM, CHEK2, or BARD1 mutation carriers. For PALB2 and ATM carriers, pancreatic cancer screening can be considered starting at age 50 or earlier if there is a relevant family history. In contrast, no additional surveillance beyond breast and ovarian screening is currently recommended for BARD1 and BRIP1 mutation carriers [28].

These guidelines represent only a starting point and are expected to be revised repeatedly as the use of multigene panels for the diagnosis of hereditary cancer becomes increasingly widespread.

In addition to its impact on screening and surveillance strategies, the broader use of multigene panel testing also carries important therapeutic implications for precision oncology. PARP inhibitors represent a class of drugs routinely used in clinical practice for the treatment of BC and OC harboring germline or somatic BRCA mutations. Moreover, they have demonstrated clinical efficacy in ovarian cancers without BRCA mutations but characterized by HRD profile, as identified by a specific biomolecular assay [33].

Recent studies have expanded our understanding of the efficacy of PARP inhibitors in tumors harboring mutations beyond BRCA1/2, including PALB2, ATM, CHEK2, BRIP1, and BARD1. Among these, PALB2 mutations have shown the most promising clinical responses to PARP inhibition [34,35,36], while the clinical benefit appears more limited for ATM [37,38] and CHEK2 variants [39]. Data on BRIP1 and BARD1 are even more exiguous and restricted to individual case reports or small exploratory studies [40,41].

Our study confirms that the application of multigene panels in the diagnostic assessment of hereditary cancer syndromes enhances the detection of causative mutations. This improvement is partly due to the identification of LP variants, which, based on in silico analyses, appeared potentially associated with the syndrome in 1.8% of cases. These findings underscore the need for regular reclassification of VUS by laboratories engaged in genetic testing, given the evolving nature of the genomic landscape.

In hereditary cancer patients, the use of comprehensive multigene panels, alongside the continuous re-evaluation of VUS and a well-defined genotype–phenotype correlation, supports accurate diagnosis and facilitates the adoption of effective cancer prevention strategies for both affected individuals and their at-risk relatives.

## 4. Materials and Methods

### 4.1. Patients

This study was carried out at the U.O.C. of Clinical and Molecular Pathology—heredofamilial Cancer, A.O.U. University of Campania “Luigi Vanvitelli” in accordance with the World Medical Association Helsinki Declaration (1964). Informed consent was obtained from all the subjects, and the study was approved and conducted according to the ethical guidelines of the University of Campania “Luigi Vanvitelli” (n.25455-07/09/2021).

We analyzed a cohort of 280 cancer patients who were referred to our clinic for suspected HBOC from June 2024 to March 2025. According to the recommendations provided by the Italian Society of Human Genetics (SIGU) [42] and the Italian Association of Medical Oncology (AIOM) [43], we selected patients based on specific clinical and familial criteria indicative of a potential HBOC syndrome. The selection criteria included individuals with a personal history of BC diagnosed before the age of 50; those with triple-negative BC diagnosed before the age of 60; patients with OC regardless of age; individuals with bilateral BC; and males with BC. In addition, patients affected by other tumor types (melanoma, CRC, lung cancer, prostate cancer, pancreatic cancer, gastric cancer) were included when the family history suggested a possible underlying HBOC syndrome (family history of breast, ovarian, pancreatic, or metastatic prostate cancer, especially when occurring in first- or second-degree relatives and at a young age).

The probands, 50 males and 230 females, received genetic counseling, and a pedigree was generated.

### 4.2. Genetic Testing

DNA from blood samples was extracted using the QIAmp^®^ DNA Blood Mini Kit (QIAGEN, Hilden, Germany) (250) according to the manufacturer’s instructions.

For mutational analysis, we used a Smart Seq Sequencing Cancer Panel on a MiSeq platform (Illumina, San Diego, CA, USA) which enables the detection of both single nucleotide variants (SNVs) and copy number variants (CNVs) through next-generation sequencing (NGS). The following genes were evaluated: BRCA1 (NM_007295), BRCA2 (NM_000059), PALB2 (NM_024675), ATM (NM_000051.4), BARD1 (NM_000465.4), BRIP1 (NM_001003694.2), CDH1 (NM_004360), CHEK2 (NM_007194), NBN (NM_002485), PTEN (NM_000314.8), RAD51C (NM_058216), RAD51D (NM_002878.3), STK11 (NM_000455.5), and TP53 (NM_000546).

The results have been interpreted from the Mutation Surveyor^®^ software, version 3.24 (Softgenetics, State College, PA, USA); ClinVar and LOVD databases were used for the identification and classification of genetic variants [44].

Sanger sequencing, as previously described [45], was used to extend genetic testing to patients’ relatives in order to assess the presence of the variant identified in the proband when feasible.

In one of the patients, we found a novel mutation in CDH1. To confirm the finding and ensure accuracy, we performed an additional sequencing run on a separate aliquot of the patient’s DNA. The mutation was identified as new by referring to the ClinVar database (https://www.ncbi.nlm.nih.gov/clinvar, accessed on 10 June 2025) and Leiden Open Variation Database, LOVD (https://www.lovd.nl, accessed on 10 June 2025). The sequence variant and mutation were named and referred in the text according to the nomenclature used by the Human Genome Variation Society, HGVS (https://hgvs-nomenclature.org, accessed on 10 June 2025).

### 4.3. In Silico Analysis

For VUS and novel variants, we performed in silico analysis.

For the missense variants, we used Rare Exome Variant Ensemble Learner (REVEL) and BayesDEL thr ough Ensembl VEP (https://www.ensembl.org/Tools/VEP, accessed on 10 June 2025); for the intronic and the 3′UTR variants, we used Combined Annotation Dependent Depletion (CADD) (https://cadd.gs.washington.edu/, accessed on 6 May 2025) and SpliceAI (https://spliceailookup.broadinstitute.org/, accessed on 6 May 2025). These tools have been largely validated in previous studies [46,47,48].

REVEL is an ensemble predictor that integrates the results of several algorithms along with evolutionary data, generating a continuous score ranging from 0 to 1. Scores closer to 1 indicate a higher probability that the variant is pathogenic, while lower values suggest a benign effect. Similarly, BayesDel uses a Bayesian model to combine functional, phylogenetic, and allele frequency data. In this case as well, the score ranges from 0 to 1, with empirically defined thresholds: scores >0.3 are generally associated with potentially pathogenic variants, while values <0.2 suggest benignity [46]. Both tools are recommended by ClinGen and the Sequence Variant Interpretation (SVI) Working Group for applying the PP3/BP4 criteria within the ACMG/AMP classification framework.

CADD calculates the score by combining different parameters derived from surrounding sequence context, gene model annotations, evolutionary constraint, epigenetic measurements, and functional predictions. For any given variant, all of these annotations are integrated into a single CADD score that ranges from 1 to 99, higher values indicating more deleterious cases [47].

SpliceAI generates four Δ scores: acceptor gain (AG), acceptor loss (AL), donor gain (DG), and donor loss (DL). These scores measure the impact of the variant on the splicing site. The Δ score ranges from 0 to 1, with higher values indicating a greater probability that the variant affects splicing. Variants with a Δ score below 0.20 are generally considered unlikely to have a significant impact on splicing, while those with Δ scores above 0.80 are typically associated with a strong impact on splicing [48].

We classified the variants according to ACMG/VCEP (https://clinicalgenome.org) into five risk categories based on the results of the bioinformatic analyses performed with the four tools described above. In particular, class I: pathogenic; class II: likely pathogenic; class III: uncertain significance; class IV: likely benign; and class V: benign. [49].

## Figures and Tables

**Table 1:** Clinical features and cancer family history of patients with PVs in BRCA1/2.

ID	Sex (M/F)	Age at Testing	Clinical Phenotype and Age at Diagnosis	Mutation Type Gene/Name and dbSNP ID	Mutation in Other Genes	Family History	Number of Family Members Tested
Patient 1	F	44	OC 44	**BRCA1:** c.116G>A (p.Cys39Tyr) rs80357498	-	Mother with PaC (42)	-
Patient 2	F	74	BC 73	**BRCA1:** c.547+2T>A rs80358047	-	Mother with OC (49) and sister with LC (65)	4
Patient 3	F	55	BC (54) and Melanoma (50)	**BRCA1:** c.4964_4982del (p.Ser1655Tyrfs*16) rs80359876	**MUTYH:** c.1103G>A (p.Gly368Asp) rs36053993	Two sisters with BC (35–46)	2
Patient 4	F	63	CRC 61	**BRCA1:** c.3756_3759del (p.Ser1253Argfs*10) rs80359876	-	Sisters with BC (45–46) and CRC (67), brother with CRC (71), and mother with BC (84) and OC (63)	1
Patient 5	F	19	BC 42	**BRCA1:** c.5266dup (p.Gln1756Profs*74) rs80357906	-	Mother with BC (60) and maternal grandmother with OC (62) and BC (63)	3
Patient 6	F	65	BC 65	**BRCA1:** c.5266dup (p.Gln1756Profs*74) rs80357906	-	Father with PC (55)	1
Patient 7	F	49	BC 49	**BRCA1:** c.5266dup (p.Gln1756Profs*74) rs80357906	-	Mother with CRC (65), sister with BC (42), and niece with BC (35)	3
Patient 8	F	18	LC 18	**BRCA1:** c.5266dup (p.Gln1756Profs*74) rs80357906	-	Mother with LC (48) and maternal grandmother with BC (65)	1
Patient 9	F	50	BC 46	**BRCA1:** c.5266dup (p.Gln1756Profs*74) rs80357906	-	Paternal aunt with CRC (69) and cousin with BC (46)	1
Patient 10	F	54	BC 54	**BRCA1:** c.5266dup (p.Gln1756Profs*74) rs80357906	-	Maternal cousin with BC (58)	1
Patient 11	F	48	BC 47	**BRCA1:** c.5319dup (p.Asn1774Glnfs*56) rs80357823	-	Maternal aunts with BC (57–67)	1
Patient 12	F	53	OC 52	**BRCA2:** c.7558C>T (p.Arg2520*) rs80357823	-	Sister with BC (50) and maternal grandmother with CRC (70)	-
Patient 13	M	40	PC 40	**BRCA2:** c.8168A>G (p.Asp2723Gly) rs41293513	-	Father with PC (65)	3
Patient 14	F	39	BC 37	**BRCA2:** c.6037A>T (p.Lys2013*) rs80358840	-	Father with CRC (61) and paternal aunts with BC (66) and LC (60)	1
Patient 15	F	64	BC 64	**BRCA2:** c.67+1G>A rs81002796	-	Father with LC (84) and paternal aunts with BC (75-35-30)	-
Patient 16	M	59	BC 59	**BRCA2:** c.67+1G>A rs81002796	-	Father with lymphoma (74) and sister with BC (42)	-
Patient 17	F	58	BC 58	**BRCA2:** c.67+1G>A rs81002796	-	Mother with BBC (80)	4
Patient 18	M	65	GC 63	**BRCA2:** c.6486_6489del (p.Lys2162Argfs*5) rs80359598	-	Mother with melanoma (68) and brother with GC (62)	2
Patient 19	F	53	BC 53	**BRCA2:** c.6486_6489del (p.Lys2162Argfs*5) rs80359598	-	Mother with BC (50) and OC (66)	-
Patient 20	M	54	BC 47	**BRCA2:** c.6486_6489del (p.Lys2162Argfs*5) rs80359598	-	Father with RCC (75) and sister with BC (47)	5
Patient 21	M	63	GC 63	**BRCA2:** c.6468_6469del (p.Gln2157Argfs*18) rs80359596	-	Mother with BC (70) and CRC (91)	-
Patient 22	F	51	BC 49	**BRCA2:** c.6468_6469del (p.Gln2157Argfs*18) rs80359596	-	Mother with BC (58) and maternal aunt with BC (69)	1
Patient 23	F	43	BC 39	**BRCA2:** c.6405_6409del (p.Asn2135Lysfs*3) rs80359584	-	Father with PC (63) and paternal uncles with PC (80) and CRC (81)	2
Patient 24	F	48	PC 48	**BRCA2:** c.5073dup (p.Trp1692fs*3) rs80359479	-	Maternal uncles with PC (70–78) and brothers with PaC (52) and PC (55)	1
Patient 25	F	89	BC 87	**BRCA2:** c.3458dup (p.Thr1154Aspfs*4) rs80359386	-	Mother with BC (70)	3

BC, breast cancer; LC, lung cancer; OC, ovarian cancer; PaC, pancreatic cancer; GC, gastric cancer; PC, prostate cancer; RCC, renal cell cancer; CRC, colorectal cancer.

**Table 2:** Clinical features and cancer family history of patients with PVs in other cancer susceptibility genes.

ID	Sex (M/F)	Age at Testing	Clinical Phenotype and Age at Diagnosis	Mutation Type Gene/Name and dbSNP ID	Mutation in Other Genes	Family History	Number of Family Members Tested
Patient 1	F	49	BC 49	**ATM:** c.3802del (p.Glu1267_Val1268ins*) rs587779834	-	Father with PC (66) and paternal aunts with PC (78) and CRC (36–85)	2
Patient 2	F	60	OC 60	**ATM:** c.3802del (p.Glu1267_Val1268ins*) rs587779834	-	Maternal aunt with BC (76) and sisters with BC (25–51), OC (37) and GC (47)	1
Patient 3	F	46	BC 46	**ATM:** c.2548G>T (p.Glu850*) rs587782280	-	Mother with BBC (50) and maternal aunts with BC (35–50) and PaC (57)	-
Patient 4	F	54	BC 53	**ATM:** c.8833_8834del (p.Leu2945Valfs*10) rs786203030	-	Mother with BC (82) and maternal aunts with BC (65-47-64)	2
Patient 5	F	53	OC 52	**ATM:** c.2250G>A (p.Lys750=) rs1137887	-	Maternal aunts with BC (80), LC (72) and sisters with LC (49) and BC (40)	1
Patient 6	F	45	BC 44	**ATM:** delEX62_EX63 (Amirifar et al., 2021) [15]	-	Paternal aunts with RCC (79), BC (65), and GC (65–67)	-
Patient 7	F	51	BC 47, TC 47, OC 45	**CHEK2:** c.920dup (p.Glu308Argfs*4) rs786203053	-	Maternal grandmother with BC (75) and sister with BC (47)	-
Patient 8	F	67	Melanoma 67	**CHEK2:** c.1427C>T (p.Thr476Met) rs142763740	-	Brother with CRC (47) and son with RCC (42)	2
Patient 9	F	30	BC 30	**CHEK2:** c.1427C>T (p.Thr476Met) rs142763740	-	Mother with BC (63), maternal grandmother with BC (65), and maternal grandfather with PC (79)	-
Patient 10	F	54	BC 54	**CHEK2:** c.1100del (p.Thr367fs*15) rs555607708	-	Maternal aunts with LC (70–75), CRC (85–88), TC (77), and PaC (77)	3
Patient 11	M	78	CRC 77	**CHEK2:** c.688G>A (p.Ala230Thr) rs748636216	-	Maternal cousin with BC (60) and daughter with OC (42)	1
Patient 12	F	52	BBC 47	**CHEK2:** c.470T>C (p.Ile157Thr) rs17879961	**MUTYH:** c.536A>G (p.Tyr179Cys) rs34612342	Mother with PaC (68)	1
Patient 13	M	59	CRC 57	**CHEK2:** c.470T>C (p.Ile157Thr) rs17879961	-	Father with GC (80) and paternal aunts with PC (78) and LC (66)	-
Patient 14	M	81	BC 81	**BRIP1:** c.478del (p.Arg160Glufs*5) rs1555616150	-	Paternal aunts with BC (50-54-60)	3
Patient 15	F	73	OC 72	**BRIP1:** c.440dup (p.Tyr147*) rs786203521	-	Maternal cousin with BC (50)	1
Patient 16	F	49	BC 37	**BRIP1:** c.55dup (p.Tyr19Leufs*2) rs1567878148	-	Mother with OC (70), maternal grandmother with CRC (72), and daughter with BC (49)	1
Patient 17	F	55	BC 55	**BRIP1:** c.55dup (p.Tyr19Leufs*2) rs1567878148	**MUTYH:** c.1187G>A (p.Gly396Asp) rs36053993	Father with LC (63) and paternal cousin with BC (38)	2
Patient 18	F	34	BC 30	**PALB2:** c.1919C>A (p.S640^*^) rs760094988	-	Maternal grandmother with BC (81)	-
Patient 19	F	39	BC 29	**PALB2:** c.1285_1286delAinsTC (p.I429SfsX12) (Vietri et al., 2015) [16]	-	Father with BC (70) and sisters with BC (32–40)	-
Patient 20	F	67	BC 43	**PALB2:** c.2257C>T (p.Arg753*) rs180177110	-	Mother with BC (60) and sister with lymphoma (39)	2
Patient 21	F	57	OC (43), BC (50), TC (50), LC (51)	**PALB2:** c.2161_2186dup (p.Ile730fs*11) rs1567217908	-	Maternal aunt OC (60)	1
Patient 22	F	46	OC 46	**RAD51C:** c.414G>C (p.Leu138Phe) rs267606999	-	Mother with OC (52) and maternal aunt with GC (72)	1
Patient 23	F	61	PaC 61	**BARD1:** c.539_540del (p.Tyr180*) rs779427628	-	Mother with PaC (69) and maternal aunt with GC (82)	3
Patient 24	M	76	LC 63	**BARD1:** c.1216C>T (p.Arg406*) rs377153250	-	Sister with BC (50) and maternal aunts with PaC (78)	-
Patient 25	F	69	BC 52	*CDH1: c.1678_1688del (p.Thr560Profs*24) ***novel***	-	Father with GC (39) and sister with BC (73) and TC (50)	1
Patient 26	M	44	GC 70	**CDH1:** c.2295+1G>C rs1596971108	-	Maternal aunt with RCC (55) and one aunt with unspecified neoplasm	2
Patient 27	F	25	BC 25	**TP53:** c.1009C>T (p.Arg337Cys) rs138398778	-	Mother with BBC (30), maternal grandmother with BBC (30), and maternal grandfather with LC (76)	-
Patient 28	M	35	PC 35	**TP53:** c.844C>T (p.Arg282Trp) rs28934574	-	Mother with BC (46)	2
Patient 29	F	40	BC 37	**TP53:** c.817C>T (p.Arg273Cys) rs121913343	-	Maternal grandmother with BC (48)	1

BC, breast cancer; BBC, bilateral breast cancer; LC, lung cancer; OC, ovarian cancer; PaC, pancreatic cancer; PC, prostate cancer; TC, thyroid cancer; RCC, renal cell cancer; GC, gastric cancer; CRC, colorectal cancer; EC. Amirifar P et al., 2021 [15]; Vietri MT et al. [16], *CDH1: c.1678_1688del (p.Thr560Profs*24): new mutation identified for the first time in our laboratory.

## Data Availability

All the data generated with this research have been included in the main manuscript.

## Supplementary Materials

The following supporting information can be downloaded at: https://www.mdpi.com/article/10.3390/ijms26135928/s1.
