# Supplementary material for: Expanding the Genomic Landscape of HBOC and Cancer Risk Among Mutation Carriers
Source: Int J Mol Sci. 2025 Jun 20;26(13):5928. doi: 10.3390/ijms26135928 (PMC12249944; doi:10.3390/ijms26135928)
Supplement: Supplementary file 1 [file ijms-26-05928-s001.zip › ijms-3676111-supplementary.pdf]

Table S1. In silico analysis of the 46 variants detected in our cohort.

Gene	Variant	dbSNP ID	Variant type	Number of patients	ClinVar and LOVD classification	Tool	Score	Prediction	Variant classification according to ACMG/VCEP criteria
**ATM**	c.4388T>G (p.Phe1463Cys)	rs138327406	missense	3	Uncertain significance	REVEL	0,758	deleterious	uncertain significance (III) BP4
						BayesDel addAF score	0,088	probably damaging	
**ATM**	c.1810C>T (p.Pro604Ser)	rs2227922	missense	5	Uncertain significance	REVEL	0,415	deleterious low confidance	likely benign (IV) BP4
						BayesDel addAF score	-0,292	benign	
**ATM**	c.2915C>T (p.Pro972Leu)	rs750093937	missense	1	Uncertain significance	REVEL	0,498	deleterious low confidance	uncertain significance (III) BP4
						BayesDel addAF score	0,326	probably damaging	
**ATM**	c.4853G>A (p.Arg1618Gln)	rs765759912	missense	1	Uncertain significance	REVEL	0,078	benign	likely benign (IV) BP4
						BayesDel addAF score	-0,358	benign	
**ATM**	c.6293T>C (p.Leu2098Pro)	rs587780631	missense	1	Uncertain significance	REVEL	0,740	deleterious	likely benign (IV) BP4
						BayesDel addAF score	0,354	probably damaging	
**ATM**	c.1960C>A (p.Gln654Lys)	rs528165789	missense	1	Uncertain significance	REVEL	0,126	benign	likely benign (IV) BP4
						BayesDel addAF score	-0,383	benign	
**ATM**	c.1516G>T (p.Gly506Cys)	rs587779816	missense	1	Uncertain significance	REVEL	0,280	benign	likely benign (IV) BP4
						BayesDel addAF score	-0,004	benign	
**ATM**	c.4871A>G (p.His1624Arg)	rs56354559	missense	1	Uncertain significance	REVEL	0,089	benign	likely benign (IV) BP4
						BayesDel addAF score	-0,291	benign	
**ATM**	c.5753G>C (p.Arg1918Thr)	rs148064985	missense	1	Uncertain significance	REVEL	0,266	benign	likely benign (IV) BP4
						BayesDel addAF score	0,016	benign	
**ATM**	c.8734A>G (p.Arg2912Gly)	rs376676328	missense	1	Uncertain significance	REVEL	0,883	deleterious	uncertain significance (III) BP4
						BayesDel addAF score	0,198	probably damaging	
**ATM**	c.1899-10T>G	rs763685190	intronic	1	Uncertain significance	CADD	15,88	not deleterious	benign (V) BP4
						SpliceAI	0,18 (AG) 0,00 (DG)	predicted not splice-altering	
**ATM**	c.8151+5G>C	rs155127370	intronic	1	Uncertain significance	CADD	20,06	deleterious	uncertain significance (III) BP4
						SpliceAI	0,00 (AG) 0,05 (DG)	predicted not splice-altering	
**CHEK2**	c.688G>C (p.Ala230Pro)	rs748636216	missense	1	Uncertain significance	REVEL	0,697	deleterious low confidance	**likely pathogenic (II) PP3**
						BayesDel addAF score	0,271	probably damaging	
**CHEK2**	c.157T>A (p.Ser53Thr)	rs371657037	missense	2	Uncertain significance	REVEL	0,170	benign	benign (V) BP4
						BayesDel addAF score	-0.018	benign	
**CHEK2**	c.1312G>T (p.Asp438Tyr)	rs200050883	missense	1	Uncertain significance	REVEL	0,337	deleterious low confidance	uncertain significance (III) BP4
						BayesDel addAF score	-0,084	benign	
**CHEK2**	c.542G>A (p.Arg181His)	rs121908701	missense	1	Uncertain significance	REVEL	0,460	deleterious low confidance	benign (V) BP4
						BayesDel addAF score	-0,155	benign	
**CHEK2**	c.319+3966G>A	rs1379926355	intronic	2	Uncertain significance	CADD	0,778	not deleterious	benign (V) BP4
						SpliceAI	0,00 (AG) 0,00 (DG)	predicted not splice-altering	
**BRIP1**	c.2440C>T (p.Arg814Cys)	rs201869624	missense	1	Uncertain significance	REVEL	0,644	deleterious low confidance	uncertain significance (III) BP4
						BayesDel addAF score	-0,069	benign	
**BRIP1**	c.550G>T	rs201047375	missense	1	Uncertain	REVEL	0,339	benign	benign

	(p.Asp184Tyr)				significance	BayesDel addAF score	-0,030	benign	(V)<br>BP4
**BRIP1**	c.2225A>C<br>(p.Tyr742Ser)	rs1555591351	missense	1	Uncertain significance	REVEL<br>BayesDel addAF score	0,910<br>0,139	deleterious<br>probably damaging	**likely pathogenic (II)**<br>**PP3**
**CDH1**	c.650C>A<br>(p.Thr217Lys)	rs778382252	missense	1	Uncertain significance	REVEL<br>BayesDel addAF score	0,678<br>0,119	deleterious<br>probably damaging	**likely pathogenic (II)**<br>**PP3**
**CDH1**	c.1009-32G>A	rs757461540	intronic	1	Uncertain significance	CADD<br>SpliceAI	7,092<br>0,00 (AG)<br>0,02 (DG)	not deleterious<br>predicted not splice-altering	benign<br>(V)<br>BP4
**CDH1**	c.1008+4del	rs746392709	intronic	1	Uncertain significance	CADD<br>SpliceAI	15,1<br>0,00 (AG)<br>0,09 (DG)	not deleterious<br>predicted not splice-altering	benign<br>(V)<br>BP4
**TP53**	c.461G>A<br>(p.Gly154Asp)	rs762846821	missense	1	Uncertain significance	REVEL<br>BayesDel addAF score	0,721<br>0,345	deleterious<br>probably damaging	**likely pathogenic (II)**<br>**PP3**
**TP53**	c.211C>T<br>(p.Pro71Ser)	rs1565556576	missense	1	Uncertain significance	REVEL<br>BayesDel addAF score	0,294<br>0,013	benign<br>benign	benign<br>(V)<br>BP4
**STK11**	c.1225C>T<br>(p.Arg409Trp)	rs368466538	missense	2	Uncertain significance	REVEL<br>BayesDel addAF score	0,238<br>-0,308	benign<br>benign	uncertain significance<br>(III)<br>BP4
**STK11**	c.121A>G<br>(p.Lys41Gln)	rs1568690070	missense	1	Uncertain significance	REVEL<br>BayesDel addAF score	0,580<br>0,146	deleterious low confidance<br>probably damaging	**likely pathogenic (II)**<br>**PP3**
**NBN**	c.643C>T<br>(p.Arg215Trp)	rs34767364	missense	1	Uncertain significance	REVEL<br>BayesDel addAF score	0,343<br>-0,1302	benign<br>benign	benign<br>(V)<br>BP4
**NBN**	c.283G>A<br>(p.Asp95Asn)	rs61753720	missense	1	Uncertain significance	REVEL<br>BayesDel	0,583<br>-0,303	deleterious low confidance<br>benign	uncertain significance<br>(III)<br>BP4
**PALB2**	c.1697G>A<br>(p.Arg566His)	rs144617793	missense	1	Uncertain significance	REVEL<br>BayesDel addAF score	0,022<br>-0,523	benign<br>benign	benign<br>(V)<br>BP4
**BARD1**	c.493A>T<br>(p.Thr165Ser)	rs876658147	missense	1	Uncertain significance	REVEL<br>BayesDel addAF score	0,182<br>-0,040	benign<br>benign	benign<br>(V)<br>BP4
**BARD1**	c.2189 A>G<br>(p.Gln730Pro)	rs876658253	missense	1	Uncertain significance	REVEL<br>BayesDel addAF score	0,349<br>0,314	benign<br>probably damaging	uncertain significance<br>(III)<br>BP4
**BARD1**	c.1973G>A<br>(p.Arg658His)	rs377227840	missense	1	Uncertain significance	REVEL<br>BayesDel addAF score	0,057<br>-0,063	benign<br>benign	benign<br>(V)<br>BP4
**RAD51C**	c.80T>C<br>(p.Leu27Pro)	rs587781309	missense	1	Uncertain significance	REVEL<br>BayesDel addAF score	0,710<br>0,376	deleterious<br>probably damaging	**likely pathogenic (II)**<br>**PP3**
**RAD51C**	c.688C>T<br>(p.Leu230Phe)	rs751528808	missense	1	Uncertain significance	REVEL<br>BayesDel addAF score	0,236<br>-0,033	benign<br>benign	benign<br>(V)<br>BP4
**BRCA2**	c.4391C>T<br>(p.Ser1464Phe)	rs587776464	missense	1	Uncertain significance	REVEL<br>BayesDel addAF score	0,02<br>-0,049	not deleterious<br>benign	benign<br>(V)<br>BP4
**BRCA2**	c.831T>G<br>(p.Asn277Lys)	rs28897705	missense	1	Uncertain significance	REVEL<br>BayesDel addAF score	0,088<br>-0,144	benign<br>benign	benign<br>(V)<br>BP4
**BRCA2**	c.3413A>T<br>(p.Gln1138Leu)	rs80358584	missense	1	Uncertain significance	REVEL<br>BayesDel addAF score	0,155<br>-0,102	benign<br>benign	benign<br>(V)<br>BP4
**BRCA2**	c.6766T>C<br>(p.Cys2256Arg)	rs559106452	missense	2	Uncertain significance	REVEL<br>BayesDel addAF score	0,537<br>-0,030	deleterious low confidance<br>benign	benign<br>(V)<br>BP4

**BRCA2**	c.8953+80G>A	rs543831222	intronic	2	Uncertain significance	CADD	0,509	not deleterious	benign (V) BP4
						SpliceAI	0,00 (AG) 0,00 (DG)	predicted not splice-altering	
**BRCA2**	c.426-126T>C	rs2072342047	intronic	1	Uncertain significance	CADD	5,147	not deleterious	benign (V) BP4
						SpliceAI	0,00 (AG) 0,00 (DG	predicted not splice-altering	
**BRCA2**	c.*50A>G	rs761312704	UTR	1	Uncertain significance	CADD	0,709	not deleterious	benign (V) BP4
						SpliceAI	0,00 (AG) 0,00 (DG	predicted not splice-altering	
**BRCA1**	c.1772T>C (p.Ile591Thr)	rs80356859	missense	1	Uncertain significance	REVEL	0,661	deleterious low confidance	uncertain significance (III) BP4
						BayesDel addAF score	0,124	probably damaging	
**BRCA1**	c.3367G>T (p.Asp1123Tyr)	rs80356867	missense	1	Uncertain significance	REVEL	0,514	deleterious low confidance	uncertain significance (III) BP4
						BayesDel addAF score	0,061	probably damaging	
**BRCA1**	c.547+68A>G	rs760491174	intronic	1	Uncertain significance	CADD	5,348	not deleterious	benign (V) BP4
						SpliceAI	0,00 (AG) 0,00 (DG	predicted not splice-altering	
**BRCA1**	c.1763_1764delinsTT (p.Ser588Ile)	rs1555591274	intronic	1	Uncertain significance	CADD	8,196	not deleterious	benign (V) BP4
						SpliceAI	0,00 (AG) 0,00 (DG	predicted not splice-altering	

Table S2. In silico analysis of the 28 novel variants identified in our cohort

Gene	Variant	Variant type	ClinVar and LOVD classification	Tool	Score	Prediction	Variant classification according to ACMG/VCEP criteria
**ATM**	c.1802+13del	intronic	not reported (novel)	CADD	2,217	not deleterious	benign<br>(V)<br>BP4
				SpliceAI	0,03 (AG)<br>0,01 (DG)	predicted not splice-altering	
**ATM**	c.8011-21T>G	intronic	not reported (novel)	CADD	9,737	not deleterious	benign<br>(V)<br>BP4
				SpliceAI	0,05 (AG)<br>0,00 (DG)	predicted not splice-altering	
**ATM**	c.8151+90G>A	intronic	not reported (novel)	CADD	0,113	not deleterious	benign<br>(V)<br>BP4
				SpliceAI	0,00 (AG)<br>0,00 (DG)	predicted not splice-altering	
**ATM**	c.2638+7A>G	intronic	not reported (novel)	CADD	3,84	not deleterious	benign<br>(V)
				SpliceAI	0,00 (AG)<br>0,00 (DG)	predicted not splice-altering	
**ATM**	c.662+42T>A	intronic	not reported (novel)	CADD	6,690	not deleterious	benign<br>(V)<br>BP4
				SpliceAI	0,00 (AG)<br>0,00 (DG)	predicted not splice-altering	
**CHEK2**	c.1260-140G>A	intronic	not reported (novel)	CADD	1,452	not deleterious	benign<br>(V)<br>BP4
				SpliceAI	0,01 (AG)<br>0,00 (DG)	predicted not splice-altering	
**CHEK2**	c.1096-104A>G	intronic	not reported (novel)	CADD	0,981	not deleterious	benign<br>(V)<br>BP4
				SpliceAI	0,00 (AG)<br>0,00 (DG)	predicted not splice-altering	
**CHEK2**	c.593-15T>G	intronic	not reported (novel)	CADD	4,104	not deleterious	benign<br>(V)<br>BP4
				SpliceAI	0,08 (AG)<br>0,00 (DG)	predicted not splice-altering	
**CHEK2**	c.1542+74A>G	intronic	not reported (novel)	CADD	5,06	not deleterious	benign<br>(V)<br>BP4
				SpliceAI	0,00 (AG)<br>0,01 (DG)	predicted not splice-altering	
**CHEK2**	c.908+32T>G	intronic	not reported (novel)	CADD	2,04	not deleterious	benign<br>(V)<br>BP4
				SpliceAI	0,00 (AG)<br>0,00 (DG)	predicted not splice-altering	
**TP53**	c.74+54G>C	intronic	not reported (novel)	CADD	5,085	not deleterious	benign<br>(V)<br>BP4
				SpliceAI	0,00 (AG)<br>0,01 (DG)	predicted not splice-altering	
**TP53**	c.993+3G>A	intronic	not reported (novel)	CADD	5,22	not deleterious	benign<br>(V)<br>BP4
				SpliceAI	0,01 (AG)<br>0,00 (DG)	predicted not splice-altering	
**PALB2**	c.109del	intronic	not reported (novel)	CADD	5,130	not deleterious	benign<br>(V)<br>BP4
				SpliceAI	0,00 (AG)<br>0,00 (DG)	Predicted not splice-altering	
**PALB2**	c.*146A>G	UTR	not reported (novel)	CADD	6.608	notdeleterious	benign<br>(V)<br>BP4
				SpliceAI	0,00 (AG)<br>0,00 (DG)	predicted not splice-altering	
**RAD51C**	c.145+117T>C	intronic	not reported (novel)	CADD	7,125	notdeleterious	benign<br>(V)<br>BP4
				SpliceAI	0,01 (AG)<br>0,01 (DG)	predicted not splice-altering	
**BARD1**	c.2189A>G<br>(p.Glu730Pro)	missense	not reported (novel)	REVEL	0.349	deleterious<br>low confidance	uncertain significance<br>(III)<br>BP4
				BayesDel addAF score	0.314	probably damaging	
**BARD1**	c.1903+24T>G	intronic	not reported (novel)	CADD	2,126	not deleterious	benign<br>(V)<br>BP4
				SpliceAI	0,00 (AG)<br>0,00 (DG)	predicted not splice-altering	
**BARD1**	c.1677+93C>T	intronic	not reported (novel)	CADD	13,06	not deleterious	benign<br>(V)<br>BP4
				SpliceAI	0,00 (AG)<br>0,00 (DG)	predicted not splice-altering	
**BARD1**	c.365-23T>A	intronic	not reported (novel)	CADD	19,6	not deleterious	benign<br>(V)<br>BP4
				SpliceAI	0,00 (AG)<br>0,00 (DG)	predicted not splice-altering	
**BARD1**	c.*27T>A	UTR	not reported (novel)	CADD	2,07	not deleterious	benign<br>(V)<br>BP4
				SpliceAI	0,00 (AG)<br>0,00 (DG)	predicted not splice-altering	
**BRCA2**	c.9649-74T>A	intronic	not reported (novel)	CADD	3,696	not deleterious	benign<br>(V)<br>BP4
				SpliceAI	0,01 (AG)<br>0,00 (DG)	predictned not splice-altering	
**BRCA1**	c.-19-109A>G	intronic	not reported (novel)	CADD	4,131	not deleterious	benign<br>(V)<br>BP4
				SpliceAI	0,00 (AG)<br>0,00 (DG)	predicted not splice-altering	
**BRCA1**	c.5152+12A>G	intronic	not reported (novel)	CADD	4,551	not deleterious	benign<br>(V)<br>BP4
				SpliceAI	0,00 (AG)<br>0,00 (DG)	predicted not splice-altering	
**PTEN**	c.165+147C>T	intronic	not reported (novel)	CADD	3,696	not deleterious	benign<br>(V)<br>BP4
				SpliceAI	0,01 (AG)<br>0,00 (DG)	predicted not splice-altering	
**PTEN**	c.803-103A>G	Intronic	not reported (novel)	CADD	3,696	not deleterious	benign<br>(V)<br>BP4
				SpliceAI	0,01 (AG)<br>0,00 (DG)	predicted not splice-altering	
**PTEN**	c.211-7-211-3del	intronic	not reported (novel)	CADD	15,78	not deleterious	benign<br>(V)<br>BP4
				SpliceAI	0,00 (AG)<br>0,00 (DG)	predicted not splice-altering	
**PTEN**	c.*106A>G	UTR	not reported (novel)	CADD	16,60	not deleterious	benign

				SpliceAI	0,00 (AG)<br>0,00 (DG)	predicted not splice-altering	(V)<br>BP4
**BRIP1**	c.93+68G>A	intronic	not reported (novel)	CADD	1,26	not deleterious	benign<br>(V)<br>BP4
				SpliceAI	0,00 (AG)<br>0,01 (DG)	predicted not splice-altering	
